# The BAL-Score Almost Perfectly Predicts Testicular Torsion in Children: A Two-Center Cohort Study

**DOI:** 10.3389/fped.2020.601892

**Published:** 2020-12-07

**Authors:** Michaela Klinke, Julia Elrod, Carolin Stiel, Tarik Ghadban, Julia Wenskus, Jochen Herrmann, Carl-Martin Junge, Konrad Reinshagen, Michael Boettcher

**Affiliations:** ^1^Department of Pediatric Surgery, University Medical Center Hamburg-Eppendorf, Hamburg, Germany; ^2^Department of General, Visceral and Thoracic Surgery, University Medical Center Hamburg-Eppendorf, Hamburg, Germany; ^3^Department of Diagnostic and Interventional Radiology and Nuclear Medicine, Section of Pediatric Radiology, University Medical Center Hamburg-Eppendorf, Hamburg, Germany; ^4^Department of Pediatric Radiology, Altonaer Kinderkrankenhaus, Hamburg, Germany

**Keywords:** testicular torsion, acute scrotum, prediction score, epidydimitis, hydatide torsion, scrotal edema, clinical

## Abstract

**Introduction:** Testicular torsion (TT) is a common emergency that warrants immediate exploration to prevent infertility or testicular loss. To improve diagnostic reliability, various scoring systems have been published. The aim of this study was to evaluate and validate different testicular torsion scores in a large cohort of children with acute scrotum.

**Methods:** Retrospective analysis of all male children that were admitted for acute scrotum at the Pediatric Surgery Department of the Altonaer Kinderkrankenhaus and University medical Center Hamburg-Eppendorf from 01/2013 to 03/2019. Two testicular torsion scores (Boettcher Alert Score, Testicular Workup for Ischemia and Suspected Torsion Score) were applied to all data sets. Furthermore, an artificial intelligence (AI)-based score was developed and compared to the two current scores.

**Results:** In total, 460 boys were included in the study. Of those, 48 (10.4%) had TT. Children with TT suffered most often from short duration of pain, nausea and vomiting, high riding testicle and absent cremasteric reflex. The BALS and the AI-based score had excellent predictive values and all patients with TT would have been detected.

**Conclusion:** The BAL and the AI score show excellent predictive capabilities and may be used to identify all cases of TT in a pediatric population. The scores are easy to apply. As the BALS was slightly better, we advocate to use this score but to validate our findings in prospective multicenter studies.

## Introduction

Testicular torsion is an immediate surgical emergency, without prompt intervention it results in an ischemia-reperfusion (IR) injury with subsequent infertility or testicular loss (1, 2). The incidence of testicular torsion in children has been estimated at 3.8% (3, 4). A recent study in 558 patients showed that early intervention (within 6 h) results in testicular salvage rate of 90–100%, whereas this rate decreases after 6 till 12 h to 20–50% and after 12 till 24 h to 0–10% (5). However, testicular torsion accounts for only a small fraction of acute scrotum cases in children (10–15%) and exploration in all children would result in unnecessary surgery in most cases (3, 6–8). Therefore, fast and definite diagnosis is essential (9).

In the past diagnosis relied heavily on clinical factors such as short symptom duration, nausea or vomiting, absence or abnormality of ipsilateral cremaster reflex, high testicle position or hard testicle (7, 10, 11). However, conclusive diagnosis is hampered by clinical overlap between the common reasons of acute scrotum (12). Hence, Doppler ultrasonography in addition to clinical examination appears to be very useful. It provides excellent sensitivity of 85–100% and specificity of 75–100% (13–15). But it is very operator dependent with a relevant risk of false-negative results (16).

To improve diagnostic reliability, by combining clinical with imaging factors, several scores have been established. Boettcher et al. defined a prediction score (Boettcher Alert Score, BALS) based on a retrospective and prospective analysis of 242 children in a center in which all children with acute scrotum were surgically explored. They identified four factors that predict testicular torsion: (1) pain duration less than 24 h, (2) nausea or vomiting, (3) high position of testis, (4) abnormal cremasteric reflex. A score of ≥ 2 was considered as high probability of testicular torsion (7, 8). Moreover, the Testicular Workup for Ischemia and Suspected Torsion (TWIST) score was described by Barbosa et al. It is based on five variables which are weighted differently: (1) swelling, (2) hard testicle, (3) nausea or vomiting, (4) high position of testis, (5) abnormal cremasteric reflex. A score of ≥3 was considered as high probability of testicular torsion and a score of ≥5 warrants immediate surgery without imaging (10, 17). Until, today only the TWIST has been validated (in a small cohort of 128 children) but not the BAL score (17). Thus, the aim of the current study was to validate and compare the two current testicular torsion scores in a very large cohort of children with acute scrotum.

## Methods

### Study Design

Retrospective cohort study of all male children (age 1–17) that were admitted for acute scrotum at the Department of Pediatric Surgery at the Altonaer Children's Hospital Hamburg (AKK) and the University Medical Centre Hamburg-Eppendorf (UKE) between January 2013 till March 2019.

### Methods

The patients were selected from the hospital database using the ICD10 codes for acute scrotum including testicular torsion, testicular appendage torsion, epididymitis and idiopathic scrotal edema. Patients suffering a testicular trauma were excluded. Additionally, patients with reduced perception of symptoms of testicular torsion due to chronic illness as well as patients with peripartal testicular torsion were excluded. Medical files, including patient charts, operating theater records and office notes, were reviewed and routinely obtained characteristics were recorded. Following items were assessed: age, duration, nausea or vomiting, position of the testicle, presence of erythema or swelling, testicle side, abdominal pain, fever, dysuria, presence of cremasteric reflex (an absent or reduced cremasteric reflex was considered an abnormal findings), tenderness of testicle/epididymis and blue dot sign as well as findings of ultrasound, serum infectious parameters, urine analysis using urine dipstick and intraoperative and microbiological findings. Physical examinations were performed by a resident and/or a senior physician of pediatric surgery and ultrasound examinations were performed by a resident of Pediatric Surgery or Pediatric Radiology.

Two different testicular torsion scores including the BALS and the TWIST score were assessed in all children (7, 8, 10, 17). In addition, an artificial intelligence (AI) based score was developed and compared to current scores (Table 1).

**Table 1:** Display of the different scoring systems for diagnosing testicular torsion, including the different weighing factors for each score.

	**BALS**	**TWIST**	**AIS**
Duration of pain <24 h	1		1
Nausea / vomiting	1	1	1
High riding testicle	1	1	2
Abnormal cremastic reflex	1	1	
Testicular swelling		2	
Hard testicle		2	
Abdominal pain			1
	2/4	3/7	2/5

### Nationwide Database Analysis

A controlled remote analysis of the nationwide DRG database from 2016 to 2017 was undertaken to evaluate inpatient data provided by the Research Data Centers of the Federal Statistical Office and the Statistical Offices of the Länder. The German adaptation of the International Classification of Diseases (ICD) Tenth Revision and the procedure coding system [Operationen-und Prozedurenschlüssel, OPS] were used to identify diagnoses and procedures. Every inpatient case with a procedure code for testicular torsion was included. Used ICD codes were N44.0, and the procedure codes were 5–622 and 5–624.

### Data Analysis

Statistics were performed using SPSS Statistics 26 (IBM, NY, USA) and R 4.0 (Foundation for Statistical Computing, Vienna, Austria). The AI-based approach was calculated using the R package randomForest (18). Random forest is a method of regression which can capture non-linear relationships by averaging the prediction of multiple decision trees (19). All features assessed in this study were utilized for the creation of the score. In terms of validation, the model was trained by randomly splitting the entire data into two parts, where 70% of the data were used for training and 30% of the data for testing. This was performed 20 times with new random distribution of the data, to eliminate outliers, and the average of the results were taken. In order to prevent over-fitting of the model to the data of the current study which could limit generalization in future real-world use, the random forest method comprises the use of different trees of which each tree is trained on a different bootstrapped dataset. In our case 200 trees were used.

The level of significance was set to 0.05.

## Results

In total 460 boys were included in the current study and 161 patients were excluded due to exclusion criteria. Due local policy all patients with elevated probability of testicular torsion underwent surgical exploration, which was nearly half of all patients presented with acute scrotum (218/460, 47.4%). In 48/460 (10.4%) patients' testicular torsion was confirmed and no testicular torsion patients was missed. The most common diagnosis was testicular appendix torsion (115/460, 25.0%), followed by epididymitis (72/460, 15.7%) and idiopathic scrotal edema (13/460, 2.8%). In 18 children no defining clinical or surgical feature was found. These cases were considered as intermitted testicular torsion (18/460, 3.9%).

Mean patient age was 9.38 (4.12) years. As shown in Table 2, children with TT were significantly older, had more often a short duration of pain (<24 h), nausea and vomiting, a high riding testicle, and an absent or reduced cremasteric reflex as well as a hard testicle than patients without TT. Erythema was significantly less common in TT than non-TT (Table 2). Swelling of the testicle, dysuria and fever was not significantly more frequent in TT or non-TT (Table 2).

**Table 2:** Clinical data for children TT compared to non-TT.

	**TT**	**Non-TT**	***P***
Age (years)	11.85 (4.29)	9.09 (4.01)	*p < * 0.001
Short duration of pain	43/48	206/412	*p < * 0.001
High riding testicle	40/48	29/412	*p < * 0.001
Nausea and vomiting	28/48	12/412	*p < * 0.001
Hard testicle	12/48	41/412	*p* = 0.006
Erythema	10/48	210/412	*p < * 0.001
Swollen testicle	33/48	234/412	*p* > 0.05
Dysuria	0/48	8/412	*p* > 0.05
Fever	0/48	4/412	*p* > 0.05

As shown in Table 3, BALS and AIS had excellent sensitivity. The BAL-Score detected all 48 cases of testicular torsion. All patients with testicular torsion showed a BAL-Score ≥ 2 (BALS ≥ 2: 48/48, BALS ≥3: 24/48, BALS 4: 8/48). Only 28 of patients without testicular torsion had a BAL-Score ≥ 2 (BALS ≥2: 28/412, BALS ≥3: 2/412, BALS 4: 0/412). Also, AI Score detected all cases of testicular torsion. An AI Score of ≥2 was reached by all 48 patients with testicular torsion (AIS ≥2: 48/48, AIS ≥3 44/48). Forty two non-TT patients also reached an AI Score of ≥2 (AIS ≥2: 42/412, AIS ≥3: 15/412). TWIST Score did not identify all cases of testicular torsion, 9 patients would have been missed (TWIST ≥3: 39/48, TWIST ≥5: 16/48). Moreover, 56 without testicular torsion had a TWIST Score ≥3 (TWIST ≥3: 56/412, TWIST ≥5: 7/412).

**Table 3:** Predictive capabilities of the current and the new score in predicting testicular torsion in the entire cohort of children with acute scrotum.

	**Sensitivity (CI 95%)**	**Specificity (CI 95%)**	**PPV (CI 95%)**	**NPV (CI 95%)**
BALS ≥ 2	100% (91.5–100%)	92.7% (91.7–92.7%)	61.5% (56.3–61.5%)	100% (98.9–100%)
BALS ≥ 3	66.7% (57.3–70.1%)	99.5% (98.4–99.9%)	94.1% (80.9–99.0%)	96.2% (95.2–96.6%)
TWIST ≥ 3	81.3% (68.2–90.3%)	86.4% (84.9–87.5%)	41.1% (34.4–45.6%)	97.5% (95.5–98.8%)
TWIST ≥ 5	66.7% (55.7–74.1%)	98.3% (97.0–99.2%)	82.1% (68.5–91.2%)	96.2% (94.9–97.1%)
AIS ≥ 2	100% (91.4–100%)	89.9% (88.8–89.8%)	53.3% (48.7–53.3%)	100% (98.9–100%)
AIS ≥ 3	91.7% (81.2–97.2%)	96.4% (95.1–97.0%)	74.6% (66.1–79.0%)	99% (97.8–99.7%)

*BALS and the AI score have perfect sensitivity with very good specificity for testicular torsion in children*.

### Nationwide Database Analysis

The nationwide database analysis revealed that in 2 years 3,374 procedures for confirmed testicular torsion were performed in Germany. Of these 443/3,374 consisted or orchiectomy without -pexie of the opposite site, 2,644/3,374 consisted orchidopexy only and finally 287/3,374 surgeries included simultaneous orchiectomy of the affected side and orchidopexy of the opposite site. Thus, in a total of 730 cases orchidectomy was necessary. Mean age was 11.20 (6.11) years and mean hospital stay was 2.19 (1.42) days. In the 2,644 children in which the testicle could be rescued (orchidopexy only) mean age was 13.25 (3.89) years and mean hospital stay was 1.56 (1.00) days. Thus, these boys in which the testis was rescued were significantly older in comparison to the boys who underwent orchidectomy (*p* <0.001) and hospital stay was significantly shorter (*p* <0.001). The above-mentioned 3,374 procedures were performed in a total of 447 centers (mean caseload per center: 7.55). Of these, 1,056 procedures were performed in low-volume institutions (1–8 procedures/2 years), and 2,318 in high-volume centers (≥9 procedures/2 years).

Complications were rare: T81 (Complications of procedures) arose in 68/3,374 cases and T88 (Other complications of surgical and medical care) occurred in 6/3,374 cases.

## Discussion

As shown by the nationwide database analysis yielding around 1,687 cases annually, testicular torsion is a very common reason for consultation. In order to prevent testicular atrophy and infertility, TT warrants immediate surgical intervention (20). Unfortunately, diagnosis may be challenging as most reasons of acute scrotum show a high clinical overlap and imaging may not be readily available (12, 21). In this current study, 460 male pediatric patients with acute scrotum that presented within 5 years were analyzed and only 10% suffered from testicular torsion. Fortunately, the BALS and AIS showed excellent predictive capabilities and none of the patients with testicular torsion would have been missed. Using the BALS, only 28 of all children in this current would have been operated upon without having TT (negative exploration rate of 6.1%), whereas the AIS would have resulted in 42 operated children without TT (negative exploration rate of 9.1%).

Even though, the BALS and AIS are similarly effective, we recommend using the BAL-Score, in particular due to its enhanced specificity in comparison to the AIS. The BALS is very easy and quick to access. It includes only four features, which are directly to access via medical history and physical examination, so no time delay is expected. Even for inexperienced residents BAL-Score is well applicable. Almost all patients with TT had (1) a duration of pain <24 h (2) a high riding testicle (3) nausea and/or vomiting and (4) absent cremasteric reflex.

It is remarkable, that while the AIS and the BALS depend more or less on the same anamnestic features plus the clinical feature “high riding testicle,” the TWIST comprises amongst others the factors “hard testicle” and “testicular swelling,” even weighing them double. In our experience evaluation of both factors can be rather complex, especially for the less experienced residents. Hence, the TWIST underperforms the AIS and BALS both in terms of sensitivity and specificity.

The combination of a clinical prediction score, e.g., BAL-Score, with ultrasonography (especially doppler US) enabled even safer diagnosis of the different reasons of acute scrotal pain, within an estimated sensitivity for identifying testicular torsion ranging between 85 and 100% (4, 22, 23). Ultrasonography become a standard imaging tool for acute scrotum, especially due to its prompt availability, fast duration of application and low costs (13). A limitation of ultrasound as diagnostic tool for acute scrotum is the high investigator dependence. Most residents are rather less experienced and a false negative US is not uncommon (16). Therefore, the application of a clinical scoring tool in combination with ultrasonography, if appropriate, will be the safest approach for a conclusive diagnosis with respect to abbreviate ischemia time of testicle.

Most limitations of the current study are inherent in a retrospective study. As in most studies that rely on clinical features, another limitation is the inter-observer variability, as experience may significantly affect the examiner's interpretation of the clinical findings (16). However, the BALS should be very robust in this regard, as the four items are easy to assess aside from the cremasteric reflex. Cremasteric reflex in adolescent patients sometimes is difficult to assess and inconclusive due to the flat response by a heavier testicle.

In conclusion, different clinical tools are available and helpful to predict testicular torsion. In our population, BAL-Score showed excellent capabilities to predict testicular torsion and reduce negative explorations to 6.1%. Due to its easy and quick application, BAL-Score is convenient for unexperienced residents as well and reduces time to conclusive diagnosis, resulting in less ischemia time.

Since, the modifications of the BAL-Score refer to the results in our cohort, prospective validation should be performed in future studies.

## Data Availability Statement

The original contributions presented in the study are included in the article/supplementary materials, further inquiries can be directed to the corresponding author/s.

## Ethics Statement

Ethical review and approval was not required for the study on human participants in accordance with the local legislation and institutional requirements. Written informed consent from the participants' legal guardian/next of kin was not required to participate in this study in accordance with the national legislation and the institutional requirements.

## Author Contributions

MK conceptualized and designed the study, acquired and analyzed clinical and experimental data, performed statistics, drafted the initial manuscript, and approved the final manuscript as submitted. JE, CS, and JW acquired and analyzed clinical and experimental data, drafted the initial manuscript, and approved the final manuscript as submitted. TG and KR acquired clinical data, drafted the initial manuscript, and approved the final manuscript as submitted. MB conceptualized and designed the study, analyzed clinical and experimental data, drafted the initial manuscript, and approved the final manuscript as submitted. All authors contributed to the article and approved the submitted version.

## Conflict of Interest

The authors declare that the research was conducted in the absence of any commercial or financial relationships that could be construed as a potential conflict of interest.
